# Sustained Resistive Switching in a Single Cu:7,7,8,8-tetracyanoquinodimethane Nanowire: A Promising Material for Resistive Random Access Memory

**DOI:** 10.1038/srep26764

**Published:** 2016-06-01

**Authors:** Rabaya Basori, Manoranjan Kumar, Arup K. Raychaudhuri

**Affiliations:** 1 Unit for Nanosciences and Theme Unit of Excellence in Nanodevice Technology, S. N. Bose National Centre for Basic Sciences, Salt Lake, JD Block, Sector III, Kolkata-700098, India

## Abstract

We report a new type of sustained and reversible unipolar resistive switching in a nanowire device made from a single strand of Cu:7,7,8,8-tetracyanoquinodimethane (Cu:TCNQ) nanowire (diameter <100 nm) that shows high ON/OFF ratio (~10^3^), low threshold voltage of switching (~3.5 V) and large cycling endurance (>10^3^). This indicates a promising material for high density resistive random access memory (ReRAM) device integration. Switching is observed in Cu:TCNQ single nanowire devices with two different electrode configuration: symmetric (C-Pt/Cu:TCNQ/C-Pt) and asymmetric (Cu/Cu:TCNQ/C-Pt), where contacts connecting the nanowire play an important role. This report also developed a method of separating out the electrode and material contributions in switching using metal-semiconductor-metal (MSM) device model along with a direct 4-probe resistivity measurement of the nanowire in the OFF as well as ON state. The device model was followed by a phenomenological model of current transport through the nanowire device which shows that lowering of potential barrier at the contacts likely occur due to formation of Cu filaments in the interface between nanowire and contact electrodes. We obtain quantitative agreement of numerically analyzed results with the experimental switching data.

In recent years a good deal of research efforts have been directed towards discovery and development of materials and devices that show electrical bias driven resistive state switching from a high resistance state (OFF state) to a low resistance state (ON state)^1^,^2^,^3^,^4^,^5^. Materials which exhibit switching from OFF state to ON state with hysteresis can be used to make memory devices like non-volatile read-only-memory (ROM)^6^,^7^,^8^. A material or a device that shows high ON/OFF current ratio, low threshold voltage for switching and large cycling endurance is desirable candidate for fabricating memory devices^5^. Resistive state switching (RSS) systems are particularly attractive for making memory elements because of their simple two terminal device structure which is able to switch the resistance of the device simply by applying a voltage or a current pulse. Nanostructured materials and nanowires (NWs) are important components in research on materials and devices that show RSS^6^,^7^,^8^,^9^. Properties of nanomaterials can engineered at nanoscopic scales which offer the possibility of integration of memory elements with large density. Comparative study indicates that the Cu:TCNQ NW device configuration presented in this report gives better performance as high density memory material in near future.

Organometallic charge transfer complexes such as Ag:TCNQ, Cu:TCNQ that show bipolar RSS, have been researched for some time as a materials for fabricating non-volatile memory^10^,^1^1. There are also proposed qualitative models that offer plausible scenario for switching in this materials. However, no direct experimental evidence or comprehensive theoretical model has so far been proposed in support of these scenarios. While past works have been done on Cu:TCNQ films or NW mats, in recent years the focus shifted to Cu:TCNQ NW array^10^,^11^,^12^. In these investigations, Cu:TCNQ NW array devices grown from Cu film with a top layer of Al/Al_2_O_3_ are used. The Al/Al_2_O_3_ electrode is generally believed to be responsible for switching^13^. However, these NW array devices typically show high threshold voltage for switching and small ON/OFF switching ratio. Table S1 is given in supplementary information, that summarizes the performance parameters of these NW array based devices.

It may be noted that Cu:TCNQ has certain advantages that makes it a natural material for making single NW based switching device. This is because Cu:TCNQ easily grows in vapor phase at rather low temperature in the NW morphology. It has been shown that it can be grown in nanobridge configuration connecting two electrodes^14^,^1^5. Interestingly the mechanisms proposed for switching in Cu:TCNQ involve formation of conductive filamentary path near contact regions by redox reaction^10^,^11^,^12^,^13^,^1^6 and tunnelling of carrier between crystalline defects^17^. These proposed mechanisms are more easily amenable in single nanowire (SNW) geometry. It has also been observed that the switching in Cu:TCNQ occurs predominantly in the contact region^13^,^1^8. Fabrication of a switching device with a SNW allows the choice of use of different contact materials that would help to maximize the performance of the switching device.

In this report, we have investigated Cu:TCNQ as a resistive switching memory material and also presented a detailed study of electrical resistive switching in Cu:TCNQ SNW devices. These devices are made from single strands of Cu:TCNQ NWs grown by vapor phase with diameter well below 100 nm the Transmission Electron Microscope (TEM) of which is shown in Fig. 1(a). Since the device is made from a single strand of NW we call it a single nanowire or SNW device. The devices were made with symmetric and asymmetric electrodes and have lateral lengths ~2 μm. SEM images of the SNW device used for measurement are shown in Fig. 1(b,c). There is an essential difference that this SNW device shows from that of previously reported devices^11^,^12^,^13^,^1^6,^18^. The devices reported here show unipolar switching in contrast to bipolar switching reported in all past reports. [N.B.: In this article unipolar switching refers to the switching where RESET (transition from low resistive state to high resistive state) process occurs for same polarity of SET (transition from high resistive state to low resistive state). Bipolar switching refers to the switching where RESET process occurs for opposite polarity of SET process. See supporting information section for more details. ]

In this paper, in addition to the experimental results on switching, we did carryout a quantitative analysis of the data to extract some of the contact parameters and to gain insight into the charge transfer kinetics that have not been done before. The analyses have been done at two distinct levels. In the first level, we do analysis of the SNW device as a metal-semiconductor-metal (MSM) device, where the NW is the semiconductor (S) and the contacts at the ends are metals (M). This allows us to separate out the contribution that occurs in the contacts as well as in the bulk of the NW. In the second level, we use a phenomenological model that is based on charge transport in the bulk of the NW and formation of metallic filaments at the contacts (as has been proposed before). The phenomenological model shows the extent of contact contribution that can change the nature of switching.

The resistance state switching with hysteresis that occur in Cu:TCNQ NWs have been proposed to arise predominantly from contacts and regions around contact. However, no quantitative measurements have been made to establish the extent of resistance switching (change) that can be attributed to changes in the bulk and that occurs in the contact region. Simultaneous measurements of 4-probe resistivity in SNW samples as well as a model analysis allow us to separate out these distinct contributions. It is to be noted that this quantitative separation is an important contribution to understand the exact mechanism of switching in this class of materials.

The observation of stable and sustainable switching with ON/OFF ratio ~10^3^ in a single strand of a Cu:TCNQ NW with diameters <100 nm connected to nanodevice platform, is a new observation. The switching experiment was carried out with stable contacts as can be used in device configuration. This observation clearly establish that if these wires are to be used in a memory device then it may be possible to work even with a single NW of diameters few tens of nanometer leading to reduction in device sizes.

This experiments reported here also establish that the bipolar resistive switching that have so far been observed in Cu:TCNQ films and NWs mats does not provide the full physical picture of switching in Cu:TCNQ NWs. The observations of unipolar switching clearly establish that the nature of switching type depends on the types of contacts.

## Results and Discussion

The 4-probe configuration in symmetric devices (see lower inset of Fig. 2) allows direct measurement of resistance of the NW used (*R*_*s*_) and its resistivity *ρ* since the dimension of the NW is known. We have measured the temperature variation of the *R*_*s*_ of the NWs used in the experiment in the temperature range 40 K ≤ T ≤ 300 K. In Fig. 2 we show the resistivity data of a 65 nm diameter NW as a function of T. The temperature dependent resistivity shows 1-dimensional variable range hopping (VRH) (*ρ*∝T^−1/2^) for T ≥ 200 K (upper inset in Fig. 2), as is expected in this type of NWs^19^. The data shown in Fig. 2 were taken with low bias, in the region where the resistivity is independent of bias. At larger bias, Cu:TCNQ shows a strong non-linear conductivity (distinct from switching) which gets enhanced significantly as the temperature is lowered. At lower T, contributions from charge density wave show up leading to substantial non-linear contribution as has been demonstrated recently^19^. For determination of *ρ* in the LRS, we measure *ρ* with a current that switches it from high resistance state (HRS) to low resistance state (LRS). The room temperature resistivity is independent of the sample diameter in the range of diameters used and is utilized as an input parameter for the device modelling discussed below. We show below that the resistivities change when the SNW device shows RSS from HRS to a LRS, although it is not the dominant factor in determining the switching ratio. The predominant change on switching occurs in the contact region. The device configuration used in the experiment measures the 4-probe resistivity in the SNW device which allows us to separate out the contribution of the NW and the contacts in switching and thus reach a clean conclusion in this point. This is in contrast to past investigation, where a 2-probe configuration used did not allow this separation.

The *I−V* curves for a typical device (marked S-1) is shown in Fig. 3. The data were obtained by sweeping up and down a dc voltage, between −5 V to +5 V. A 10 kΩ load resistor was placed in series with the sample to protect the NW from current surge that occurs at the switching. The *V* value shown is the bias across the device. Initially NW device was in HRS or OFF state. With increasing bias current increases (arrow ‘1’) and at the threshold voltage *V*_*SET*_, the SNW device rapidly switches from an OFF state or HRS to ON state or LRS, indicated by the sharp increase of current in the circuit (arrow ‘2’). The device current after switching reaches the instrumental compliance limit as shown in Fig. 3. The RSS which leads to a transition from HRS to LRS at *V*_*SET*_ has ON/OFF ratio of nearly 10^3^ in the particular device shown. We define the ON/OFF ratio as the ratio of the device currents in LRS and HRS on switching. With reducing applied bias, the device current reduces (arrow ‘3’), but does not follow the path that it takes while switching to ON state. On further reduction of bias the device turns back to HRS from LRS at a bias ~0.25 V. This has the same polarity as the *V*_*SET*_, but has much less value. We refer to this as the reset voltage (*V*_*RESET*_). Since SET and RESET process occurs for same polarity of applied bias we refer to it as unipolar switching. This behavior is qualitatively different from the bipolar switching behavior observed earlier in Cu:TCNQ films and nanobridges^11^,^12^,^13^,^1^6. where the SET and RESET occur at opposite polarities.

Similar electrical behaviour is also observed under reversed polarity of applied bias, but with lower ON/OFF ratio although the electrodes are made from nominally the same materials. The change in ON/OFF ratio in positive and negative polarity is due to difference in contact areas of deposited C-Pt with the NW at the two ends as well as difference in the barrier potentials.

The resistive state switching observed in the Cu:TCNQ SNW device is stable. We have collected data continuously for few days. Figure 4 shows switching data (ON/OFF ratio, *V*_*SET*_ and *V*_*RESET*_ ) for 450 successive cycles for device S-2. Data shown are for every 10 cycles. This graph shows that stable and reproducible switching of unipolar nature can be obtained in a SNW with sub-100 nm diameter. At the end of large number of cycles (more than 1000) the devices show instability which we analysed as arising from charge stored in the electrode regions. Thus, such a large cycling endurance with high ON/OFF ratio and low threshold voltage will supposed to make Cu:TCNQ SNW a promising material for ReRAM integration.

In this paper, we present results taken on two symmetric devices which differ mainly on the details of the contact characteristics. The second device (marked S-2) is made from a NW with smaller diameter (65 nm). The device shows very similar behavior and *V*_*SET*_ and *V*_*RESET*_ values, but has somewhat smaller ON/OFF ratio. The relevant numbers are collected in Table 1.

**Figure 1:**
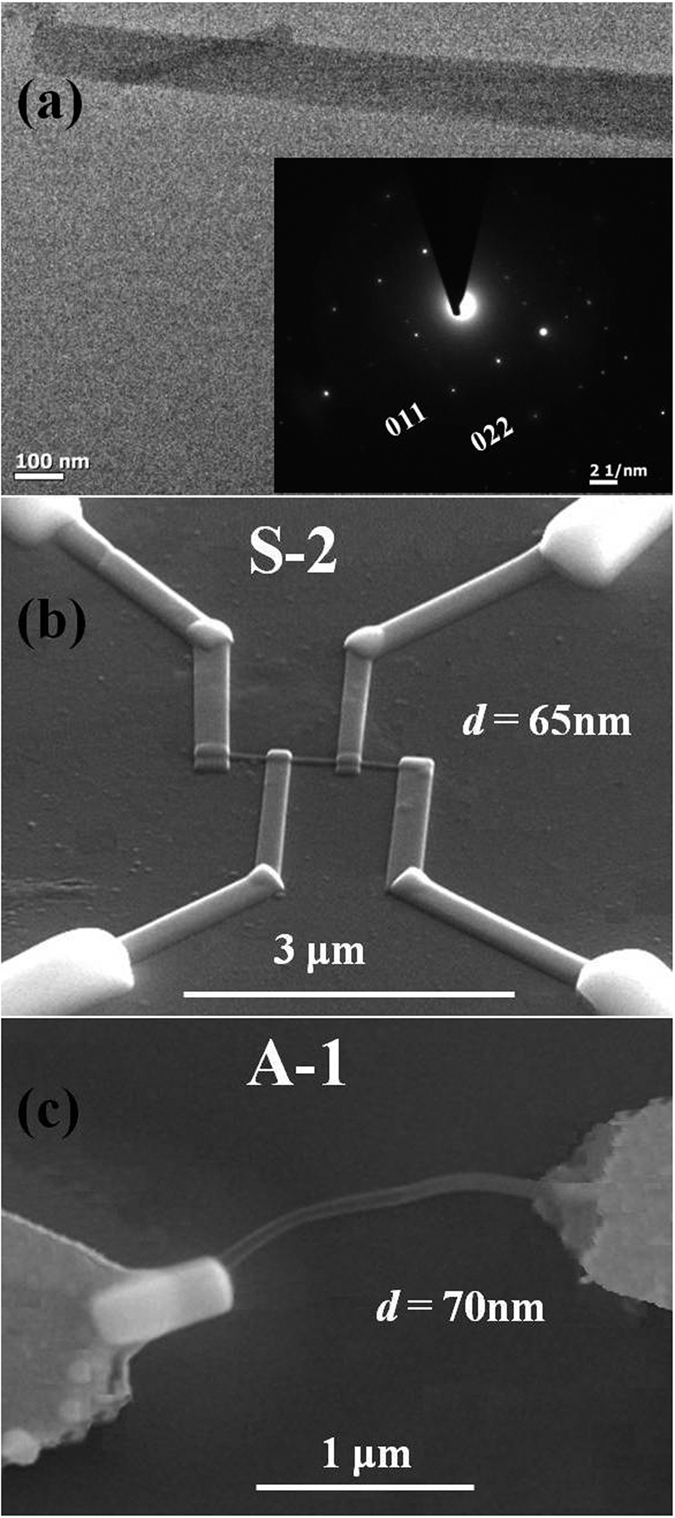
Structural characterization of Cu:TCNQ nanowire by TEM and SEM images of the devices. (**a**) TEM image of a SNW device. Inset shows the SAED patterns of the same. SEM image of PVD grown Cu:TCNQ NW devices having (**b**) symmetric (C-Pt/Cu:TCNQ/C-Pt) and (**c**) asymmetric (Cu/Cu:TCNQ/C-Pt) electrode configuration.

In Fig. 5 we show switching data taken on SNW device (A-1) with asymmetric electrodes as shown in Fig. 1(c). The asymmetric device has the configuration Cu/Cu:TCNQ/C-Pt. This device also shows unipolar resistive switching and transition from HRS to LRS occurs at threshold voltage of *V*_*SET*_ = 3.3 V (arrow ‘2’). Reverse state (LRS to HRS) occurs at *V*_*RESET*_ ~ 0.7 V. The ON/OFF ratio achieved is in the vicinity of 10^4^ similar to that has been obtained in the symmetric device S-1. In this case the switching is observed only in one polarity i.e. when C-Pt electrode of the NW is in negative polarity w.r.t. the other electrode. (In symmetric devices described in previous sub-section, one of the C-Pt electrodes is always reversed bias, while the other electrode is forward biased. As a result switching is observed in both polarities of bias although with different ON/OFF ratios). From the results of devices with symmetric and asymmetric electrodes it is clear that the nature of electrodes plays an important role in switching of the SNW device which is discussed below extensively. The observed threshold voltages *V*_*SET*_ in both types of SNW devices (symmetric and asymmetric) are lower than those seen in RSS in other reported devices of Cu:TCNQ array^11^,^1^3 or SNW^12^,^1^7.

The results presented above clearly demonstrate that it is possible to get reproducible and robust RSS in Cu:TCNQ SNW devices with diameter of the NW down to at least ~60 nm. The typical lengths of the devices studied are in the vicinity of ~2 μm. These devices differ from past devices made on Cu:TCNQ films or NW arrays which have relatively much large structures. Both types of devices studied showed unipolar switching in contrast to past reports that showed bi-polar RSS. There are some reports on RSS in a SNW of Cu:TCNQ^12^,^1^7 connected between two Au electrodes. Zheng el al.^17^ observed switching in a Cu:TCNQ SNW of diameter 400 nm at a threshold voltage of 34 V and ON/OFF ration ~10^4^ and was explained by field-assisted inter-stack interaction mechanism, but did not provide clear evidence of that. Xiao *et al.*^12^ observed switching in a SNW of diameter 20 nm with ON/OFF ration ~10^2^ at a threshold voltage of 9 V. Although they explained switching with redox reaction leading to formation of Cu filaments, but no detailed investigation has been done. In both the cases there is lack of information about nature of switching i.e. it is unipolar or bipolar. (See Table S1 in supporting information section).

The results reported here clearly show that contacts play an important role in the switching phenomena. In particular, the results on asymmetric device establish that switching occurs when the contact with a barrier is reversed biased. Past investigations indicated the role of contact on bipolar switching observed in planar devices made on Cu:TCNQ films. These investigations also localized the cause of switching near the contacts^13^,^1^6,^18^. However, the role of contact that has been discussed in past studies is qualitative in nature and no quantitative model based analysis was done which allows evaluation of actual changes in contacts as well as that in the body of the NW.

In this section, we present two models for analysis of the data and provide a quantitative evaluation to the data analysis that explains our observations. The models are developed in two levels. In the first model, we use a simple approach of a MSM device that allows us to obtain quantification of contacts parameters (potential barriers parameters at the contacts and the changes that occur to the barriers on switching). This analysis allows us to evaluate an important issue, whether any change occurs in the resistance of the NW when the HRS to LRS state occurs. Since we can evaluate the resistivities of the NWs in HRS as well as LRS by 4-probe resistivity measurements, we can separate out these contributions. In the next model, we use a phenomenological approach that is based on a microscopic model for switching. The model used is physically similar to that proposed before to explain bipolar switching in devices like Cu/Cu:TCNQ/Al^13^,^1^8. However, we add quantitative steps into the model and numerically reproduce the observed switching data, in particular, the unipolar switching data. In this model, using a rate equation approach we explore the development of Cu filaments at the contact region that eventually lead to lowering of potential barriers.

The Cu:TCNQ SNW devices that have been investigated here are 2-terminal devices with two metallic electrodes (M) connecting the semiconductor (S) NW. This type of 2-terminal devices can be thought of as MSM devices^20^,^21^,^22^. We have used such a device model to analyze the opto-electronic data on SNW photodetectors made from Cu:TCNQ NWs^14^,^2^3. However, analysis of the switching data in Cu:TCNQ SNW devices using MSM model has not been done before. We show that the model gives us a clear indication of the role of the contacts that lead to large switching from a HRS to a LRS. The model allows us to separate out the contributions that occur due to the changes in the barrier heights at the contacts as well as that due to resistance change in the NW. In case of bipolar switching, the phenomena widely studied in Cu:TCNQ NW array, the model of the switching mechanism that have been proposed, is based mainly on modification at the contact^13^,^1^8,^24^,^25^. However, it has never been established quantitatively the contributions coming out from the contacts and that from NW. We show below that the unipolar switching reported here has contributions from both the processes. The dominant contribution arises from contact modifications, where the switching leads to substantial change in the barrier heights in the contact region.

The conventional MSM device model has been developed for a semiconductor device with barriers (e.g. Schottky barriers) at the contacts^20^,^21^,^22^. The Cu:TCNQ SNW devices that have configuration C-Pt/Cu:TCNQ/C-Pt can be considered as symmetric MSM devices (albeit with unequal barrier heights) and devices with Cu/Cu:TCNQ/C-Pt configuration are asymmetric MSM devices where one contact is ohmic. Strictly, the contacts on the Cu:TCNQ are not conventional Schottky type contacts. Nevertheless, it can be reasonably assumed that there is a contact with barrier at metal-semiconductor junction so that a MSM type model can be used to get quantitative information on relevant parameters. The contacts can be considered as regions with barriers that need an applied bias to have a transport through it. The barrier height in the present treatment is a lumped parameter that describes the activated nature of the transport at the contacts.

The MSM model uses contacts connected by the NW resistance (*R*_*s*_). This makes one of the contacts forward biased and other reversed biased, which exchange when the bias polarity is reversed. The switching leads to change of *R*_*s*_ as well as the barriers. The MSM model gives the following equation for the *I-V* curve as^26^


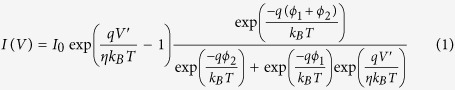


where, *V′* = *V* − *IR*_*s*_, *R*_*s*_ being the series resistance arising from the NW and *ϕ*_*1*_ and *ϕ*_*2*_ are the barrier heights associated with the two contacts (M’s) in forward and reverse bias respectively, *k*_*B*_ being Boltzmann constant, *η* is the ideality factor and *I*_*0*_ is current flow across the metal-semiconductor interface. In the symmetric devices we can measure the exact value of *R*_*s*_ from the 4-probe resistivity measurements in HRS (OFF) state as well as in LRS (ON) state. This allows us to get a direct validation of the parameters, because *R*_*s*_ obtained from the fit using the equation above can be compared with the directly measured values. It is noted that the two values of *R*_*s*_ obtained by fit procedure and direct measurements agree to within ± 5–10%, which is a direct validation of the model used.

The fits to the MSM model are shown in Fig. 6 for the two devices; S-1 and A-1. It can be seen that good fits to the data can be obtained for both polarities where the device current changes by few orders of magnitude. The parameters obtained from the fits are summarized in Table 2. The resistance values of the NWs used in the MSM fit are obtained from the measured resistance data. The table also shows the change in the barrier heights (*ϕ*_*1*_ and *ϕ*_*2*_) at switching. Table 1 show the ON/OFF resistance ratios of the different samples measured. The reductions observed in barrier heights on switching quantitatively establish the primary changes that actually occur in the contact parameters on switching.

The following important inferences can be obtained from the MSM fit:

(a) There is a change in resistance (*R*_*s*_) of the NWs after switching, although the extent of change (factor of 5–6) is much less than the orders of magnitude change that occur in the device current at the switching. This establishes essential role of the contact. This change in resistance in the body of Cu:TCNQ NW is similar to that arising from non-linear conductance in Cu:TCNQ NW recently observed^19^. The observed magnitude of change in *R*_*s*_ may depend on nature of contact electrodes that determine the value of the exact potential drop across the length of the sample.

(b) The *ϕ*’s in the HRS in the NW/C-Pt contacts range from 0.2 eV to 0.47 eV. This is similar to that observed before for SNW optical detectors where C-Pt contacts were used before^26^. The variability of the values of *ϕ*’s arise from four main factors: namely, the exact C and Pt content in the deposit; actual area of the contact; surface states on the Cu:TCNQ NWs and the exact extent of charge transfers between the Cu and TCNQ NW in the region where the contacts are made. Presence of more Cu in the electrode region will definitely lower the barrier value. For the Cu/Cu:TCNQ/C-Pt asymmetric device the value of *ϕ* at the Cu contact from which the NW grow is ~0.02 eV. This being energetically less than the thermal energy at 300 K (0.025 eV), this contact acts as an ohmic contact and does not contribute to switching. For this device, C-Pt contact has a relatively high barrier value ~0.47 eV. This arises mainly due to the way this contact has been made on the NW touching the top Au electrode (see Fig. 1(c)). Switching occurs when this contact is reversed biased.

(c) The contact barrier heights undergo significant changes on switching as can be seen from Table 2. On switching the reversed biased contact undergoes most changes. Such reduction of barrier value at reverse polarity (i.e, cathode) is taken as the evidence of formation of metallic channel at this junction. The switching ratio in this model is directly determined by the lowering of the barrier heights. For device S-1 where largest ON/OFF ratio has been observed in switching, the barrier with *ϕ*_*1*_ = 0.37 eV undergoes a reduction by nearly 0.23 eV, which is ~60% of the original value. Since the barrier values occur in exponential this leads to a large change in the device current.

To summarize this subsection, using a simple MSM device model it is found that the switching occurs primarily due to lowering of contact barrier at the cathode (the reversed bias junction) with additional contribution arising from lowering of resistance of the NW, although with less effect. The lowering of the contact potential agrees well with qualitative mechanisms suggested before where formation of Cu filaments at contacts has been suggested as the cause of switching. Lowering of the resistance of the NW on switching is manifestation of non-linear conductance in the NW, because on switching a much larger current flows through it.

In this sub-section, we develop a simple model based on rate equation of charge transport in the SNW device. The schematic of the model is shown in Fig. 7 which presents a qualitative scenario of the unipolar switching. The model/scenario depicted below draws its motivation from earlier qualitative models^13^,^1^8,^24^,^25^ proposed for bipolar switching in Cu:TCNQ, in which redox reaction leads to filament formation (Cu-filament) at contacts that take part in the switching process. In this model, Cu:TCNQ NW acts as a source of Cu^+^ ions. When sufficient bias is applied across the NW, Cu^+^ ions migrate from NW to the negative polarity junction through the interface and are reduced to Cu atoms. As bias increases this process continues and with time filament like structure forms between NW and C-Pt electrodes junction which reduces the barrier height to a very low value facilitating charge transport. The sudden change in barrier height results in sharp jump in current at *V* ≥ *V*_*SET*_. Reduction of the bias reduces the current and when bias reaches to *V*_*RESET*_, aligned Cu-filaments are disrupted due to oxidation of Cu-filaments because of higher electro-negativity of Pt-C electrodes. The oxidation of Cu-filaments leads to ionic Cu or localized electronic states and, therefore, there is enhancement of barrier height resulting in reduction of current to low value and this brings the system back to HRS. The nature of switching (unipolar) reported here differs from the previously reported switching (bipolar) data. Nanowire device with Cu/Cu:TCNQ/Al configuration shows bipolar switching, whereas device with Cu/Cu:TCNQ/C-Pt or C-Pt/Cu:TCNQ/C-Pt exhibits unipolar switching. The reason can also be well explained by considering the electron affinity (EA) of the electrodes material connection the NW and ionization potential (IP) of the NW itself. Contact leads with low electron affinity (like Al) gives bipolar, whereas electrodes with high electron affinity (like Pt) gives unipolar type of switching. [For more details see *Appendix III* in the supporting information].

We develop the above qualitative scenario into a phenomenological model below based on rate equations that describe the charge transport in the device. The SNW device in this model is divided into two contact regions at the ends (‘*l’* and ‘*r’*) and a central part (‘*c’*) that is the body of the NW. In the contact region the transport will be tunnelling type through metallic islands and also thermionic emission where the metal at the end makes contact with NW. The charge transport in the SNW device depends on the following parameters: (a) the transition rates ‘*Г* ’ between sites, (b) occupation number or fractional filling ‘*n’* of the systems, (c) the number of states ‘*N’* involved in the processes, (d) energy gap or potential barrier ‘*ϕ* ‘at the ends and (e) applied voltage ‘*V’*. The body of the NW (referred as ‘*c*’) and the contact regions at left (‘*l’*) and right (‘*r’*) differ in the values of the fill fractions *n*’s. The rate equations below describe the changes in *n*’s. Let us consider the electrodes are half-filled systems. The Cu:TCNQ have a partial charge transfer from Cu to TCNQ which stacks in such a way that it forms a one dimensional π-stacking structure. Cu is present in Cu:TCNQ mostly as Cu^+^ ion that has charge transferred with TCNQ. Generally the charge transfer index, Z ≈ 0.5–0.6 as measured. It would imply that about half of the sites in which Cu can go are filled and since there is empty orbital available in Cu^+^, an electron injected from the electrode that is reversed bias can go to the empty sites. Population of an electron on these Cu^+^ sites will reduce theion sites will reduce the ions making them Cu which will lead to formation of Cu filaments. Similarly, if electrons are taken away from it in the forward biased electrode the Cu^+^ sites will reduce theions will get oxidized to Cu^2+^ sites will reduce thenear the same electrode. Using the language of fill fraction then in initial condition, when we have mostly Cu^+^ sites will reduce theions we can assume all *n*’s are equal and ≈0.5. As the current passes, near the forward bias electrode *n* will increase and near the reverse biased electrode *n* will decrease due to reduction of Cu^+^ sites will reduce theto Cu. Therefore, the contact region with reduced Cu forms filaments when *n*^*l*^ or *n*^*r*^ decreases from 0.5.

We propose a rate equation similar to the rate equation developed for explaining switching in oxides^27^,^2^8. Based on the above assumptions and controlling parameters, a rate equation for the electron transport can be written for the SNW device as follows:


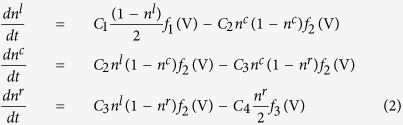


where, *n*^*l*^, *n*^*c*^ and *n*^*r*^ are occupation at left contact region, center NW region and right contact region respectively. *C*_*1*_, *C*_*2*_, *C*_*3*_ and *C*_*4*_ are the transition rates constant from left electrode to left contact, left contact to NW, NW to the right contact and right contact with right electrode respectively. *C*_*x*_ are proportional to *T*_*x*_*D*_*l*_*D*_*r*_, where *T*_*x*_ depends on scattering matrix element from state |I〉 to | *f*  〉^29^, *D*_*l*_ and *D*_*r*_ are density of state on the left and right side material of the junction. The derivation of current at a junction is explained in the supplementary section. For simplicity, we can define the bias dependent barrier transfer function *f* (*V* ) as


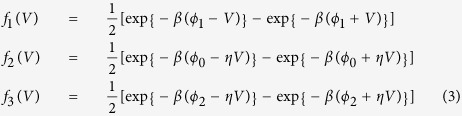


Here, *ϕ*_*1*_, *ϕ*_*0*_ and *ϕ*_*2*_ are the potential barrier height from the left electrode of left contact region, potential barrier between contacts to the NW and right contact and right electrode respectively. *η* is a fraction of voltage drop because of resistance and capacitance of the contact region. *ϕ* depends on the filament formation and goes to smaller value when all Cu^+^ is reduced to Cu and goes up in case of Cu^+^ oxidation at low *V* < 0.5 eV.

The current in the device is proportional to the electron current from electrode to contact region, 
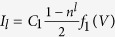
 or 
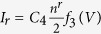
. The calculated *I−V* curves are shown in Fig. 8 for two devices with different electrode configurations (S-1 and A-1). In the numerical evaluations, it was assumed that the contact regions are half-filled initially so that *n*^*l*^ = *n*^*r*^ = 0.50 and *n*^*c*^ = 0.48, which makes the NW region semi-conducting. The appropriate choice of parameters (*C*’s) can reproduce the calculated curve that mimics the measured curves. For the HRS the values of the barriers used for numerical evaluation are those obtained from the experimental data using MSM model. For reproducing the observed curves we use, *C*_1_ = 9 × 10^−3^, *C*_2_ = 2 × 10^−4^ and *C*_3_ = *C*_4_ = 1.8 × 10^−4^. *ϕ*_0_ = 0 and experimental values of *ϕ*_1_ and *ϕ*_2_ for LRS and HRS are used as shown in Table 2. The shape of the curves is very sensitive to values of *C*. As shown in Fig. 8, the jump in |*I*_*l*_| curve occurs at V ≈ 3.5 V where *n*^*l*^ goes to 0.5 and systems achieve LRS. It remains in this phase till it reaches to *V* = 0.5 V. For *V* < 0.5, electronegativity of the electrodes wins and withdraws electrons from the contact region to oxidise the Cu-filament. Therefore, the system back to HRS. In the reverse bias, a similar phenomenon occurs.

In the device A-1, the values of *C* are *C*_1_ = 1.8, *C*_2_ = 2.5 × 10^−5^ and *C*_3_ = *C*_4_ = 2 × 10^−4^ respectively. The large values of *C*_*1*_ can be justified on the basis of the ratio of amplitude of current on forward bias and reverse bias is close to 1, but potential barrier is almost 20 times higher. As shown in Fig. 8(b) at *V* = 3.5 V barrier heights *ϕ*_*1*_ changes from 0.47 eV to 0.36 eV, whereas *ϕ*_*2*_ remains constant as give in Table 2 because system goes from HRS to LRS. Again, this transition is attributed to reduction of Cu^+^ at the contact and the *n*^*l*^ = 0.5 at this voltage. We notice that there is no a significant jump on the reverse bias as *ϕ* is very small and does not change significantly.

The physical mechanism that changes the system from HRS to LRS is the barrier height reduction at the electrodes interface in reverse bias condition. The barrier height reduction is caused by the formation of metallic channel by redox reaction at that interface. Thus, in LRS condition electronic states are more aligned compared to that in HRS. This aligned state remains unaffected until as formed metallic channels break at lower bias (*V*_*RESET*_). This causes hysteresis in *I−V* in the device.

## Conclusion

In conclusion, an extensive investigation (experimental as well numerical modeling) has been undertaken on SNW switching devices that have been fabricated on Cu:TCNQ NW with sub-100 nm diameter. The devices have symmetric (C-Pt/Cu:TCNQ/C-Pt) and asymmetric (Cu/Cu:TCNQ/C-Pt) electrode configurations. These devices exhibit unipolar switching with ON/OFF ratio approaching 10^4^ and relatively low *V*_*SET*_ ≈ 3.5 V and a much lower *V*_*RESET*_ ≈ 0.1 × *V*_*SET*_, although past reports show bipolar switching in these types of NWs or films. The switching in the SNW device was found to be robust with reproducible switching parameters like ON/OFF ratio, *V*_*SET*_, *V*_*RESET*_ and cycling endurance gives signature of promising future material for ReRAM. It is noted that there are very few reports of resistive switching in single Cu:TCNQ NW and extensive experimentation as well as model based quantitative data evaluation have not been done.

We have applied MSM model to analyse the *I−V* data from which we could establish that the principal source of switching is at the contacts. The switching was found to be caused by large drop of contact potential in reverse biased contacts and are not seen when the contact is ohmic or has a low barrier.

The 4-probe device configuration of the symmetric SNW device allowed us to obtain the resistivities of the NWs in the ON (HRS) and OFF (LRS) states directly which was not reported before. It has been found that although the switching leads to lowering of resistivity of the NW by a factor ~5, this is much less than the switching ratios (ON/OFF ratios). The change in the resistivity of the NW may not be considered as a switching state transition but more like a non-linear conductivity that occurs due to large current flowing through the device.

A numerical analysis based on a phenomenological model of charge transport through the NW (along with contacts) has been developed which shows that the charge transport leads to change in fractional occupation of sites (fill fraction) at the contact region as the applied bias is gradually increased upto the *V*_*SET*_. This was interpreted as a signature of formation of Cu filaments at the reversed biased contacts that leads to lowering of contact potential barrier giving rise to large change in device current and switching. The transition to OFF state from the ON state occurs when the applied bias is reduced leading to reverse change of site fraction fill factors that eventually leads to break down of Cu filaments by thermal factors at the active contact leading to upward swing of the contact barrier potential and large reduction in the device current at *V*_*RESET*_ that leads eventually transition to OFF state.

## Materials and Methods

### Growth of Cu:TCNQ nanowires

The SNW switching devices were fabricated from Cu:TCNQ NWs grown by vapour phase deposition of TCNQ onto Cu. The charge transfer reaction of TCNQ with Cu leads to formation of Cu:TCNQ and growth of NW that involves diffusion of Cu through the growing Cu:TCNQ NW. The Cu that diffuses along the length of the NW acts as a fresh supply source for further reaction and growth^15^. The NWs grown were characterized using X-Ray Diffraction (XRD), Transmission Electron Microscope (TEM) and Fourier Transform Infra-red (FTIR) spectroscopy. Briefly, the XRD data established that the Cu:TCNQ NWs have tetragonal structure and belong to the higher conductive phase-I^30^. The TEM data along with selective area electron diffraction (SAED) pattern established the single crystalline nature of the NWs. TEM image of a single Cu:TCNQ NW, of the type used in this work, is shown in Fig. 1(a). FTIR was used to establish the extent of charge transfer in Cu:TCNQ from Cu to TCNQ. The details of growth and characterization of the charge transfer complex NWs are given elsewhere^19^,^3^0.

We performed electrical RSS measurements in two types of SNW devices. This allows investigation of the effect of electrodes connecting the NW on switching. In both types of devices, the diameter of the NWs ranges from ~65 nm to ~90 nm. In one class of devices we use the same material to make the electrodes which we refer to as the symmetric device. In a second class of device, the two electrodes are of dissimilar metals. We refer to such a device as an asymmetric device. As described below, the NWs used for the symmetric devices were made separately on a Cu film (*ex-situ*) and then integrated on to the device in 4-lead configuration. In case of an asymmetric device, the wire is grown on the Cu electrode (*in-situ*) and connected to the other electrode in a 2-lead nanobridge configuration. Table 3 summarizes the dimensions of different devices used for measurements.

### Fabrication of SNW devices with symmetric electrode

SEM image of a SNW device with symmetric electrodes is shown in Fig. 1(b). The NWs used were grown on a Cu film by physical vapor deposition (PVD) of TCNQ as described in the subsection before. For attaching leads, synthesized NWs were dispersed from a suspension in etahnol on a Si_3_N_4_ (300 nm thick)/Si (insulating) substrate containing pre-fabricated Au/Cr (100 nm/10 nm) electrodes which were used as contact pads. The operation of attaching leads to individual NWs was done in a dual beam machine FEI HELIOS 600. The NWs were imaged during fabrication using the built-in FE-SEM (Field-Emission Scanning Electron Microscope) of the dual beam machine. After selection of a particular NW, four Pt leads were connected to the NW to make the SNW device.

**Figure 2:**
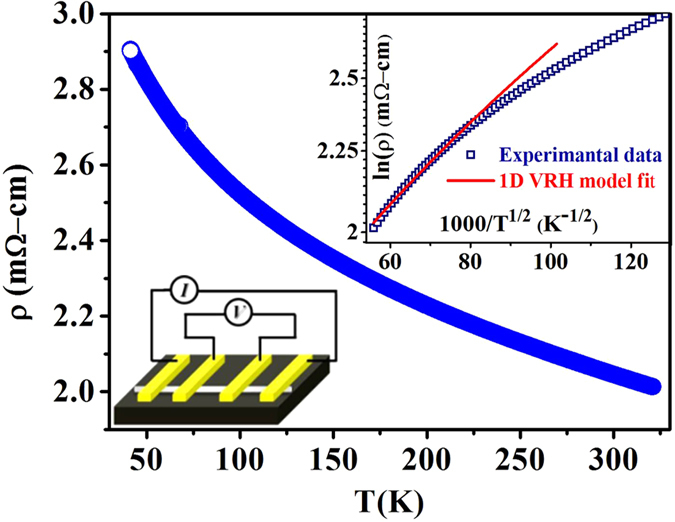
Temperature dependent resistivity of Cu:TCNQ SNW device of diameter 65 nm. (Upper inset) 1-dimensional VRH model fit for T ≥ 200 K. (Lower inset) Schematic of device configuration used for measurement.

**Figure 3:**
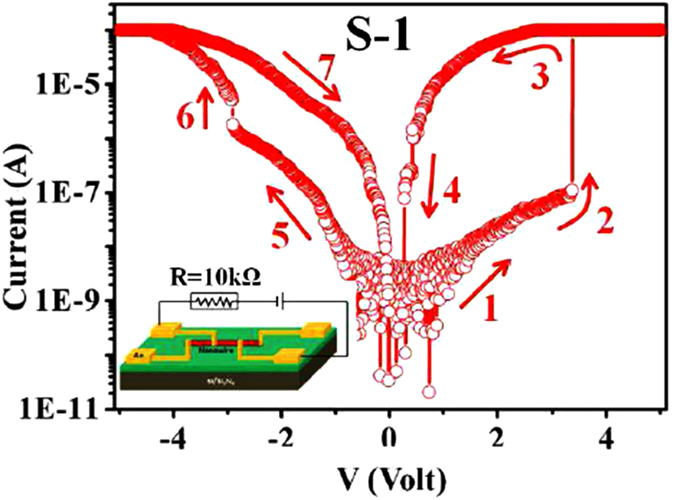
Log *|I|*−*V* curves of Cu:TCNQ SNW devices with diameter 90 nm connected with FEB deposited C-Pt in C-Pt/Cu:TCNQ/C-Pt symmetric electrode configuration. Series load resistance R = 10 kΩ and measurement has been performed in air (inset).

**Figure 4:**
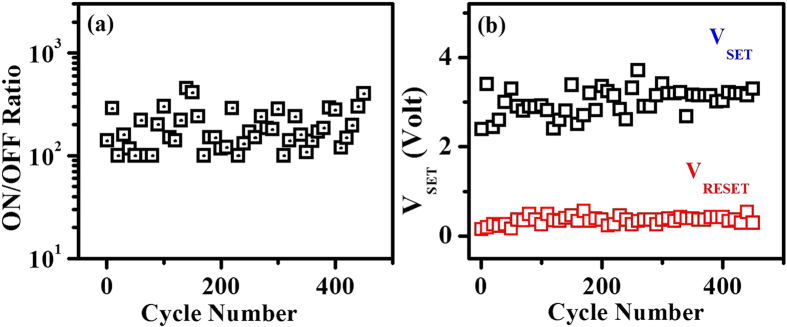
Large cycling endurance and low threshold voltage. (**a**) ON/OFF current ratio and (**b**) threshold voltage of the device S-2 as a function of cycle number.

**Figure 5:**
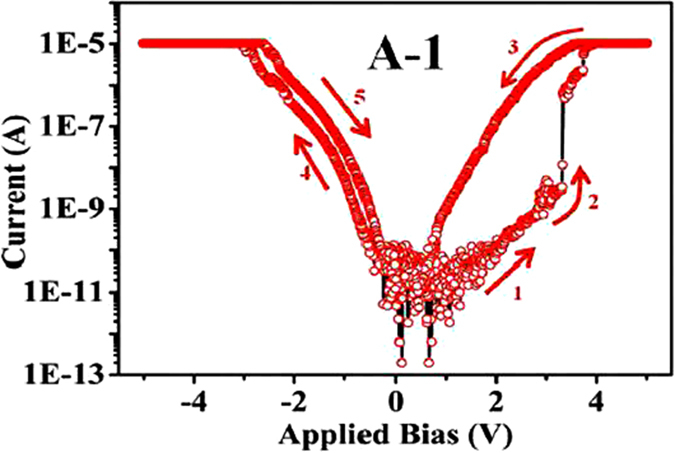
Log *|I|*−*V* curves of a SNW of Cu:TCNQ in Cu/Cu:TCNQ/C-Pt asymmetric electrode configuration measured in air. *I−V* shows unipolar switching only for one polarity of the applied bias when connecting end of the nanowire is negatively biased.

**Figure 6:**
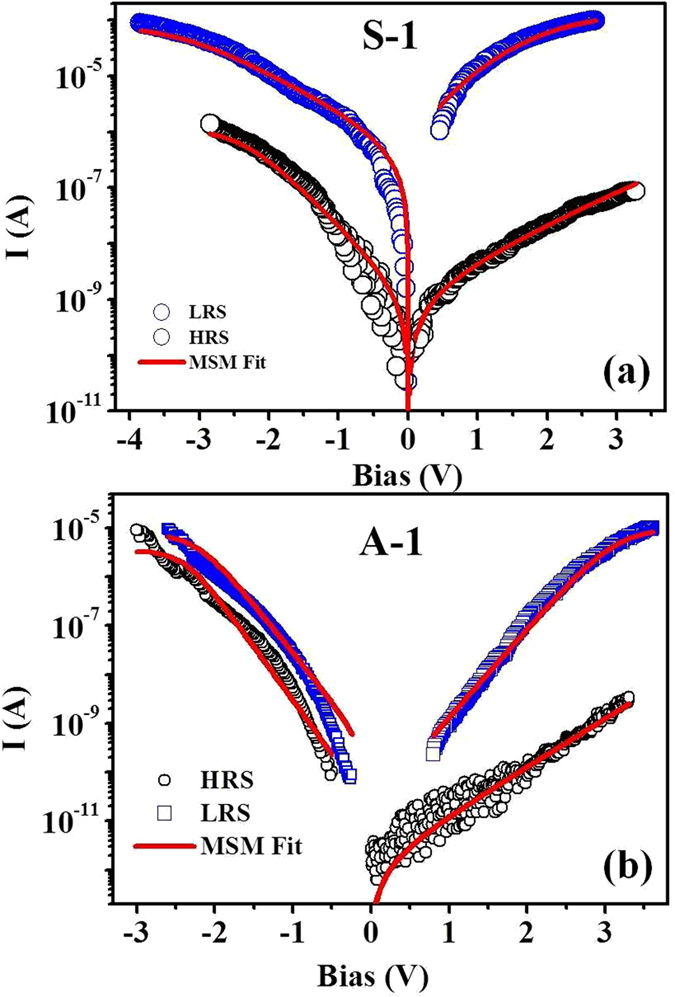
MSM fit for a, symmetric and b, asymmetric electrode configuration. Here, FEB deposited C-Pt acts as metal (M) and Cu:TCNQ NW as semiconductor (S).

**Figure 7:**
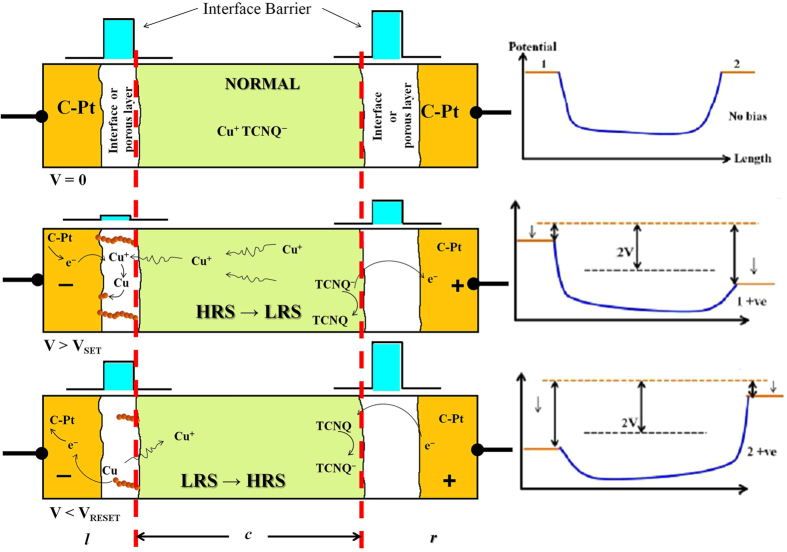
Schematic of phenomenological model considered. Cu:TCNQ NW is sandwiched between to two electrodes made up of FEB deposited C-Pt, separated by two interface region where filament formation occurs. *‘l’* represents electrode-NW junction with reverse bias, ‘*r*’ is electrode-NW junction with forward bias and ‘*c*’ is NW.

**Figure 8:**
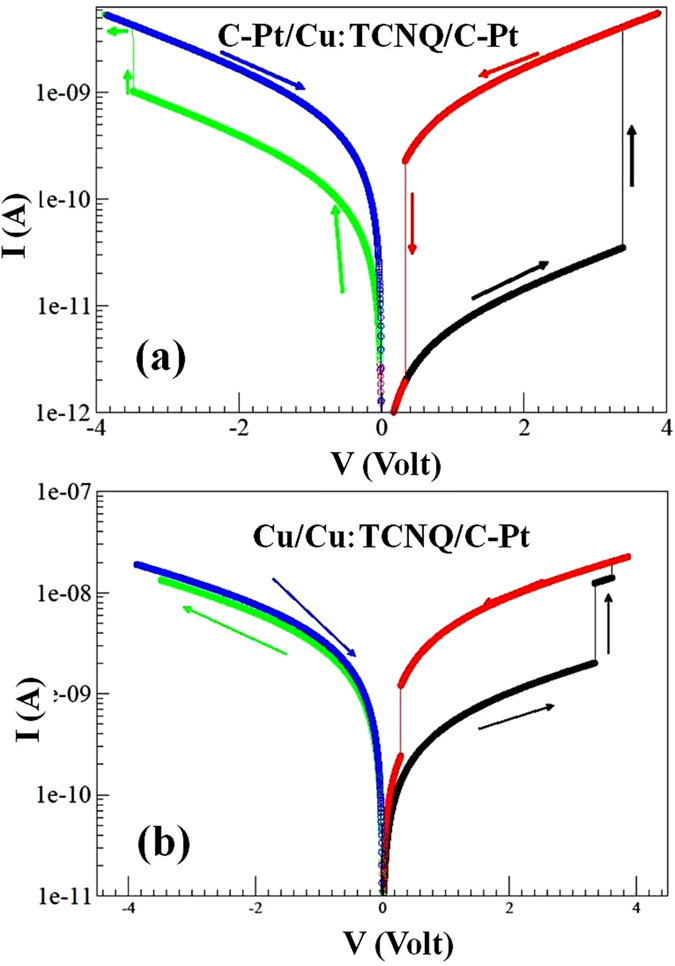
Results obtained from numerical analysis using Eqn. (2) for (**a**) symmetric (S-2) and (**b**), asymmetric (A-1) electrode configuration. Simulated results are well supported by the experimental results.

Deposition of Pt was done by focused electron beam induced deposition (FEBID) using the precursor Methylcyclopentadienyl platinum trimethyl C_5_H_4_CH_3_Pt(CH_3_)_3_. It is noted that Pt electrodes grown by FEBID is a composite of amorphous carbon (C) majority phase and a dispersed phase of Pt.^31^. Due to the composite nature of the electrode, it is referred to as C-Pt. Data have been presented here on two symmetric devices S-1 and S-2. Final device configuration is referred as ‘C-Pt/Cu:TCNQ/C-Pt’. These devices are prepared with 4-probes as shown in Fig. 1(b) allowing us determination of the resistances (and hence resistivities) of the NWs directly without contribution from contact resistance. Two outer probes in the SNW devices were used for RSS measurements (see lower inset of Fig. 2). It needs to be clarified that the device is referred as symmetric because both electrodes are made from the same material (C-Pt). This does not imply that the two electrodes have identical characteristics like the same barrier heights. This difference will be clarified in a subsequent section where we discuss analysis of the data.

### Fabrication of SNW device with asymmetric electrode

The SNW devices with asymmetric electrodes were fabricated by growing the NWs in a nanobridge configuration with prefabricated tri-layer electrodes on insulating substrate of Si/Si_3_N_4_^14^,^3^2. The tri-layer electrodes of Cr/Cu/Au (~10 nm/50 nm/100 nm) were prepared by e-beam lithography and lift-off. While Cr acts as the adhesion layer, the Cu acts as the growth layer from which the growth of Cu:TCNQ NW originates when TCNQ vapor is exposed to it. An additional thick Au layer (~100 nm) on top of Cu prevents the TCNQ vapor from reacting with the upper surface of the Cu layer and thus limit the growth of Cu:TCNQ NWs to the sides of the electrodes. This *in-situ* method of growth and fabrication of the SNW device is distinct from that discussed in the previous sub-section where *ex-situ* synthesis was performed to fabricate the SNW device. Details of nanobridges growth are given in earlier publications by the authors^14^,^1^9.

SEM image of the SNW device, marked as A-1 is shown in Fig. 1(c). Free end of the NW is anchored to the top Au with FEBID of Pt. The final device has the configuration ‘Cu/Cu:TCNQ/C-Pt’. Device dimension is stated in Table 3. The device is said to be asymmetric as growth end of the NW is pure Cu which acts as an ohmic contact while the other end anchored with C-Pt acts as a contact with a finite barrier. It will be shown below that this asymmetric nature of the contact shows up directly in the switching behavior of the device.

## Additional Information

**How to cite this article**: Basori, R. *et al.* Sustained Resistive Switching in a Single Cu:7,7,8,8-tetracyanoquinodimethane Nanowire: A Promising Material for Resistive Random Access Memory. *Sci. Rep.* **6**, 26764; doi: 10.1038/srep26764 (2016).

## Supplementary Material

Supplementary Information

## Figures and Tables

**Table 1:** Switching voltage and ON/OFF ratio of different devices.

Device	NW Diameter (nm)	V_SET_ (Volt)	V_RESET_ (Volt)	ON/OFF Ratio	
				+ve polarity	−ve polarity
S-1	90	3.35	0.4	10^3^	10^2^
S-2	65	3.53	0.4	10^2^	10^2^
A-1	70	3.3	0.7	10^4^	1

**Table 2:** MSM fit parameters for I−V curves.

Device	HRS(+)			LRS(+)			HRS(−)			LRS(−)		
	*R*_*s*_ (kΩ)	*ϕ*_*1*_ (eV)	*ϕ*_*2*_ (eV)	*R*_*s*_ (kΩ)	*ϕ*_*1*_ (eV)	*ϕ*_*2*_ (eV)	*R*_*s*_ (kΩ)	*ϕ*_*1*_ (eV)	*ϕ*_*2*_ (eV)	*R*_*s*_ (kΩ)	*ϕ*_*1*_ (eV)	*ϕ*_*2*_ (eV)
S-1	24	0.372	0.200	4	0.135	0.021	24	0.200	0.372	4	0.026	0.150
S-2	75	0.342	0.204	2	0.236	0.023	75	0.242	0.360	2	0.018	0.242
A-1	45	0.488	0.021	9	0.360	0.023	45	0.026	0.338	9	0.028	0.286

Here ***R***_***s***_ is known parameter calculated from 4-probe resistivity measurement both in HRS and LRS.

**Table 3:** Electrode configuration and dimension of the devices.

Device	Electrode Configuration	Dimension	
		Length (μm)	Diameter (nm)
S-1	Symmetric (C-Pt/Cu:TCNQ/C-Pt)	1.5	90
S-2	Symmetric (C-Pt/Cu:TCNQ/C-Pt)	2.5	65
S-3	Asymmetric (Cu/Cu:TCNQ/C-Pt)	1.9	70
