# Supplementary material for: Sustained Resistive Switching in a Single Cu:7,7,8,8-tetracyanoquinodimethane Nanowire: A Promising Material for Resistive Random Access Memory
Source: Sci Rep. 2016 Jun 1;6:26764. doi: 10.1038/srep26764 (PMC4887791; doi:10.1038/srep26764)
Supplement: Supplementary Information [file srep26764-s1.doc]

***Supporting Information***

**Sustained Resistive Switching in a Single Cu:7,7,8,8-tetracyanoquinodimethane Nanowire: A Promising Material for Resistive Random Access Memory**

Rabaya Basori, M. Kumar, and A. K. Raychaudhuri

**Appendix I**

Table S1: Comparative study of switching parameters in charge transfer complex nanowire.

| **System** | **Nanowire diameter (nm)** | **Threshold voltage (V)** | **I_ON_/I_OFF_**  **ratio** | **Reference** |
| --- | --- | --- | --- | --- |
| Cu:TCNQ SNW | 20 | Set-7, Reset-10 | 10 | *Adv. Mater. 18, 2184(2006)* |
| Cu:TCNQ SNW | 400 | 34 | ~10^4^ | *NML, Vol-1, p23-26 (2009)* |
| Cu:TCNQ ANW | 50 | Set-10, Reset-7 | 20 | *APL, 96,263504 (2010)* |
| Cu:TCNQ ANW | 60-200 | 5.5 | 6x10^3^ | *Nanotech. 19, 015305(2008)* |
| Ag:TCNQ SNW | 80 | 7.5 | ~10^4^ | *Adv.Mater.Sci.5 (2003) 72-75* |
| Ag:TCNQ SNW | 60 | 8.5 | 10 | *Adv. Mater. 21, 4742–4746 (2009)* |
| **Cu:TCNQ SNW** | **70** | **3.3** | **~10^4^** | **This work** |
| **Cu:TCNQ SNW** | **90** | **3.3** | **>10^3^** | **This work** |

**Appendix II**

**Types of resistive switching**

Resistive switching refers to the physical phenomena where a dielectric suddenly changes its (two

terminal) resistance under the action of an electric ﬁeld or current. A schematic of switching is shown

in Fig. 5.3 When a certain voltage or current is applied across the device, it goes from high resistance

(OFF) state to the low resistance (ON) state by application of a threshold voltage called SET voltage

(VSET) at certain polarity, whereas for same or opposite polarity of SET voltage the system returns to its high resistive or OFF state and the corresponding voltage is called reset threshold voltage (VRESET). Depending on the polarity of SET and RESET voltage, resistive switching can be classiﬁed into two categories: unipolar and bipolar. [Ref: R. Waser; Nanoelectronics and Information Technology, Wiley - VCH, 2003]

1. **Unipolar switching**

Unipolar devices are switched from the high resistance (OFF) state to the low resistance (ON) state

(SET operation) by application of a threshold voltage (SET). The electrical current must be limited to a compliance current (CC) during the set process in order to prevent the device from instantly returning to the OFF state, or even worse the possible memory cell destruction. This is due to the fact, that the SET and RESET state can take place at the same polarity. The reset operation occurs at a higher current and a lower voltage than the set threshold voltage. Schematic diagram of unipolar switching is shown in Fig. S1 (a).


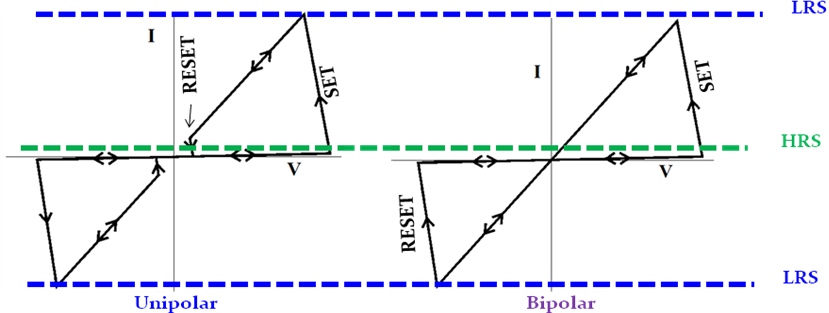


Figure S1. Schematic representation of (a) unipolar and (b) bipolar electrical resistive switching. HRS indicates for High Resistive State, and that of LRS is for Low Resistive State.

1. **Bipolar switching**

Bipolar devices are switched from the high resistance (OFF) state to the low resistance (ON) state by

application of a threshold voltage. The current is often limited by a current compliance during the

ON switching operation in order to prevent a possible memory cell destruction. The RESET operation

takes place at the opposite polarity by application of a different threshold voltage. The MIM or MSM

structure of the system must have some asymmetry (different electrode materials, electroforming step,

etc.) in order to show bipolar switching behavior. Schematic diagram of bipolar switching is shown in

Fig. S1 (b).

**Appendix III**

**Reason behind unipolar and bipolar switching:**

It has been observed that a type of switching i.e. it is unipolar or bipolar depends on the nature of electrodes connecting the nanowires. Nanowire device with Cu/Cu:TCNQ/Al configuration shows bipolar switching, whereas device with Cu/Cu:TCNQ/C-Pt or C-Pt /Cu:TCNQ/C-Pt exhibits unipolar switching. The dependence of the switching behaviour on the electrodes can be explained based on electron affinity (EA) and ionization potential (IP) of the electrode and the Cu filaments. EA and IP are listed in the table S2. To break the copper filament Cu should go from Cu (metallic) state to Cu^+2^ state. The electron transfer depends on the IP of Cu and EA of electrode. Therefore, E=IP(Cu)-EA(electrode) is a relevant quantity. We notice that E for Pt and Al electrodes are ~ 2.21 and ~ 0.46 eV. Hence, ionizing the Cu filament with Pt electrode is much easier than with the Al electrodes. As shown in Fig. S2 (a), negative bias is applied at Al electrode and electron moves from Al to Cu^+2^ ions which in turn from metallic Cu-filaments between Al electrode and Cu-TCNQ salts at V_SET_= -3.5V. Henceforth, systems go to a LRS. The arrangement remain intact till the potential of Al reach to V=3.5V and afterwards, electrons start flowing back to Al and Cu gets ionized and filaments breaks down. In second case, where C-Pt is electrode interfacing with Cu, Pt and C both have very IP as shown in table S2 and E of this Pt-Cu interface is 1.6 eV smaller than previous interface. As shown in Fig S2 (b), negative bias is applied to the Pt electrodes, electrons move from Pt to Cu ion and Cu-filaments formation takes place, but other side of the interface region gets the electron from Cu-TCNQ. Therefore, we need a threshold voltage V_SET_ = 3.5 V to form the Cu-filaments in interface regime. While decreasing the potential, at V_RESET_ = 0.5 V and below this potentially Cu-TCNQ pull the electron to from filament to bring the systems in HRS. Similar phenomenon occurs in the negative bias.


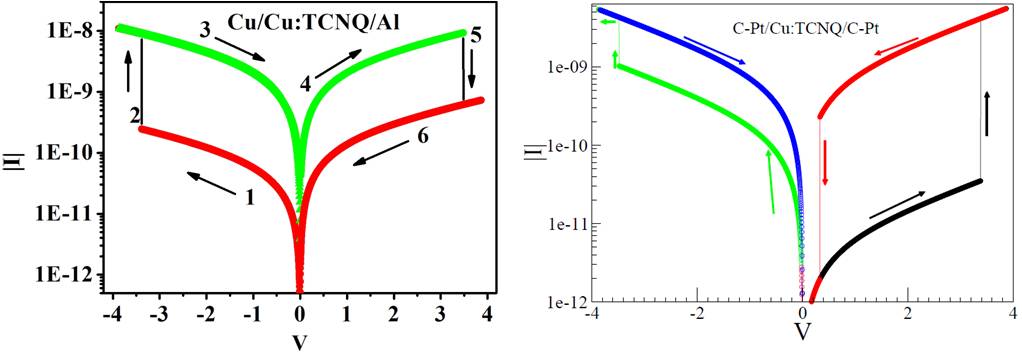


Fig S2: Simulated results of (left) bipolar electrical resistive state switching in Cu/Cu:TCNQ/Al device configuration, whereas C-Pt/Cu:TCNQ/C-Pt device configuration (right) shows unipolar switching.

**Table S2: The electron affinity (EA) and Ionization Potential (IP)**of different elements.

| Material | Ionization Potential (eV) | Electron Affinity (eV) |
| --- | --- | --- |
| C  Al  Cu  Pt  TCNQ  O_2_ | 11.26  5.98  7.72  9.01  9.6  13.6 | 1.26  0.46  1.22  2.12  2.81  1.46 |

Cu:TCNQ is a conductor having both electronic and ionic conduction through metallic (Cu) and insulating (TCNQ) channels. Cu:TCNQ acts as source of Cu^+^ and TCNQ^−^ ions. When –ve bias is applied across Pt electrode. Cu^+^ ions migrate from Cu:TCNQ NW through the interface of NW and Pt to the Pt electrode. Electron affinity of Cu being lower compared to Pt, Cu takes electron from the source and reduced to neutral Cu atoms. This process continue with time and forms metallic filament between NW and Pt –ve electrode at V=V_set_, which takes the system to LRS. When bias at Pt reduces to very low value TCNQ being highly electronegative, residual TCNQ molecules inside the NW takes electron from metallic Cu and filaments break. This brings the system to HRS and it exhibits unipolar switching.

In Cu/Cu:TCNQ/Al configuration, a thin AlOx layer is assumed to be formed by oxygen adsorption at the interface between the CuTCNQ layer and Al top electrode. This model suggest that the switching occurs by the migration of Cu+ ion through a thin porous oxide layer at the CuTCNQ/oxidizable electrode interface when Al electrode is –vely biased. As bias reduces, oxygen being highly electronegative, it prevents to oxidize Cu atom at negative bias and hence filaments are intact. But, for a sufficiently +ve bias at Al electrode interface, i.e. by reversing the polarity the metallic Cu is electrochemically oxidized into Cu+ cations and the filaments dissolves, leading to the HRS. This in this configuration, transition from HRS to LRS occurs for one polarity and reverse process occurs for opposite polarity, exhibiting bipolar switching.

**Appendix IV**

**Derivation of rate equation using Fermi-Golden rule:**

Let us consider a junction of two material and represented as left (*l*) and right ( *r* ). The scattering strength Γ of electron from a left E_l_ state to a right E_r_ state can be given by Fermi-Golden rule and the expression of Γ written as

(S1)

Where f(E) is the Fermi function and |T­_lr_| is hopping matrix element between the state E­_l_ and E_r_ . The scattering amplitude depends on the number of occupancy of left and vacancy of right *i.e.* electrons from all the occupied states of left can scatter to right side unoccupied states. Therefore, the general Γ can be written [Ref: Book: David, K. Ferry and Stephen, M. Goodnick, *Transport in Nanostructures, Cambridge University press*, 1997.] as

(S2)

Let us assume that the (E_l_ –E_r_ ) is large, the Fermi-function can approximated f(E)~e^-β(E^_l_ ^– E^_r_ ) n_l_ (1-n_r_ )[Ref: *Phy. Rev. Lett.* **92**, 178302−4 (2004); *Appl. Phy. Lett.* **88**, 033510−3 (2006)]. Therefore, Eq.2 can be written as

$\Gamma^{l\to r}=An_{l}(1-\mathrm{n}_{r})e^{-\beta(E_{l}-E_{r})}$ (S3)

Now the current can be defined as

(S4)

Here, φ is barrier height of the system considering the left side Fermi-energy as a reference point. C is proportional to density of states; therefore it depends on the structure and type of atom of material. C also depends on the hopping matrix element |T| between states of different materials as shown in Eq. S1. T depends on the overlap of wave function ie the distance between closest atoms of materials at the junction. In this device there are three different parts and we can write three equations as in Eq. 2. Each equation has two parts; incoming current is first term and second term is outgoing current.
